# Editorial: Memory T Cells in Chronic Infections and Tumors

**DOI:** 10.3389/fimmu.2021.656010

**Published:** 2021-02-17

**Authors:** Maike Hofmann, Camilla Jandus, Lian Ni Lee, Daniel T. Utzschneider

**Affiliations:** ^1^Clinic of Internal Medicine II (Gastroenterology, Hepatology, Endocrinology and Infectious Diseases), Freiburg University Medical Center, Faculty of Medicine, University of Freiburg, Freiburg, Germany; ^2^Department of Pathology and Immunology, Faculty of Medicine, University of Geneva, Geneva, Switzerland; ^3^Ludwig Institute for Cancer Research, Lausanne, Switzerland; ^4^Nuffield Department of Medicine and Translational Gastroenterology Unit, Peter Medawar Building, University of Oxford, Oxford, United Kingdom; ^5^Department of Microbiology and Immunology, The Peter Doherty Institute for Infection and Immunity, University of Melbourne, Melbourne, VIC, Australia

**Keywords:** exhaustion, memory T cells, chronic infection, tumor, PD-1

Immunological memory by T cells is a fundamental characteristic of the adaptive immune system. The underlying principle is that exposure to a pathogen leads to the generation of long-lived memory T cells, which provide an immediate and stronger immune response following re-encounter of the same pathogen, ensuring effective protection following a pathogen reinfection or vaccination (1). While pathogenic eradication and thus re-establishment of quiescence has been assumed to be a prerequisite for the formation of immunological memory, recent advances in the field have revealed that T cells with memory-like characteristics can be found among exhausted T cell responses in ongoing infections or in tumors (2). As such, these cells, distinguishable by the expression of the transcription factor T-cell factor 1 (TCF1) or the chemokine receptor CXCR5, retain proliferative potential and the ability to self-renew while also continuously replenishing the pool of antigen-specific exhausted effector T cells, thereby mediating viral or tumor control (3, 4). Interestingly, TCF1+ memory-like or precursor T cells can also exhibit features of T cell exhaustion, as described for many actively-persisting viral infections such as Human Immunodeficiency Virus (HIV) (5, 6) and Hepatitis B or C viruses in humans (7, 8), Lymphocytic choriomeningitis virus (LCMV) infections in mice (3–5, 9), and in tumor settings both in humans and mice (10–13). Most importantly, TCF1+ T cells are responsible for the boost of immunity following immune checkpoint blockade in immunotherapy (3, 4). Thus, promoting our understanding of the molecular characteristics of these memory-like or precursor T cells will drive the design and development of novel immunotherapies to modulate exhausted T cell responses in chronic infections and tumors.

This Research Topic was developed to encourage high-quality scientific outputs on this particular theme. As such, the Research Topic comprehensively highlights in two review articles as well as three primary research articles the importance of memory-like or precursor T cells in chronic infections and tumors. First, Martinez-Usatorre et al. unequivocally identified and established an improved cell sorting protocol to isolate TCF1+ precursor T cells from solid tumors. The authors performed transcriptomic analysis of PD1+ T cells derived from murine B16 tumors as well as primary human melanoma tumors to identify an enriched expression of *Slamf6* by concomitant lack of *Entpd1* (encodes CD39) and *Havcr2* (encodes TIM3) expression among precursor T cells. Based on these expression patterns, they demonstrate that PD1+Slamf6+CD39-TIM3- tumor-infiltrating lymphocytes (TILs) displayed higher proliferative potential when isolated, transferred, and rechallenged, compared to the PD1+Slamf6-CD39+TIM3+ counterpart. Thus, Martinez-Usatorre et al. nicely demonstrate a viable approach to improve the isolation of superior TILs that can strongly advance efficacy of adoptive cell therapy.

Zhang et al. addressed the role of the inhibitory receptor CD160 on CD8 T cells responding to chronic viral infection. The authors first demonstrate that CD160 expression positively correlates with improved clinical signs such as enhanced CD4 T cell counts and reduced viral burden in people with HIV. Moreover, the authors utilize the preclinical chronic LCMV model to elegantly demonstrate that CD160 is required to maintain prolonged viral control. Importantly, the authors reveal that the lack of viral control is due to an impaired generation of TCF1+ precursor T cells in the absence of CD160 and thus an impairment in sustaining a long-term T cell response. Thus, signaling through CD160 is essential for the formation of TCF1+ precursor T cells in response to chronic viral infection.

Kumashie et al. set up a mouse model to study T cells responding to persistent antigen presented by hepatocytes. By doing so, the authors identify exhausted intrahepatic precursor T cells and demonstrate that these cells retain proliferate and self-renewal potential similar to splenic precursor T cells responding to chronic viral infections. Interestingly, the authors demonstrate for the first time that precursor T cells retain improved metabolic and mitochondrial potential compared to their CXCR5- counterpart. This is an important observation that addresses a central topic of how T cell exhaustion and T cell metabolism are regulated and connected. Li and Zhang dive into this field by reviewing the current literature of cellular metabolism of different memory T cell subsets and highlighting different possibilities to target mitochondrial metabolism to boost T cell memory formation and metabolic fitness to improve cancer immunotherapy.

Finally, the review by Nüssing et al. takes a step back and addresses specific similarities and discrepancies of T cell exhaustion and tolerance. The authors discuss how tumors can manipulate tolerance mechanisms to evade immune recognition.

Overall, this Research Topic contains a range of articles that highlight the critical role of memory-like or precursor T cells across a wide range of diseases emphasizing the importance to further improve our understanding of memory T cell biology to design new or improve the efficacy of current immunotherapies.

## Author Contributions

All authors contributed equally in writing the manuscript.

## Conflict of Interest

The authors declare that the research was conducted in the absence of any commercial or financial relationships that could be construed as a potential conflict of interest.
